# New Coumarins and Anti-Inflammatory Constituents from the Fruits of *Cnidium monnieri*

**DOI:** 10.3390/ijms15069566

**Published:** 2014-05-28

**Authors:** Tzong-Huei Lee, Yuan-Chih Chen, Tsong-Long Hwang, Chih-Wen Shu, Ping-Jyun Sung, Yun-Ping Lim, Wen-Lung Kuo, Jih-Jung Chen

**Affiliations:** 1 Graduate Institute of Pharmacognosy, Taipei Medical University, Taipei 110, Taiwan; E-Mail: thlee@tmu.edu.tw; 2 Department of Pharmacy & Graduate Institute of Pharmaceutical Technology, Tajen University, Pingtung 907, Taiwan; E-Mail: cute88883333@yahoo.com.tw; 3 Graduate Institute of Natural Products, College of Medicine, Chang Gung University, Taoyuan 333, Taiwan; E-Mail: htl@mail.cgu.edu.tw; 4 Department of Medical Education and Research, Kaohsiung Veterans General Hospital, Kaohsiung 813, Taiwan; E-Mail: cwshu@vghks.gov.tw; 5 National Museum of Marine Biology and Aquarium, Pingtung 944, Taiwan; E-Mail: pjsung@nmmba.gov.tw; 6 School of Pharmacy, College of Pharmacy, China Medical University, Taichung 404, Taiwan; E-Mail: limyp@mail2000.com.tw; 7 Chung-Jen College of Nursing, Health Science and Management, Chiayi 600, Taiwan; E-Mail: m049@cjc.edu.tw

**Keywords:** *Cnidium monnieri*, umbelliferae, coumarins, structure elucidation, anti-inflammatory activity

## Abstract

The fruit of *Cnidium monnieri* is commercially used as healthcare products for the improvement of impotence and skin diseases. Three new coumarins, 3'-*O*-methylmurraol (**1**), *rel*-(1'*S*,2'*S*)-1'-*O*-methylphlojodicarpin (**2**), and (1'*S*,2'*S*)-1'-*O*-methylvaginol (**3**), have been isolated from the fruits of *C. monnieri*, together with 14 known compounds (**4**–**17**). The structures of these new compounds were determined through spectroscopic and MS analyses. Compounds **1**, **4**–**12**, and **14**–**17** exhibited inhibition (IC_50_ ≤ 7.31 µg/mL) of superoxide anion generation by human neutrophils in response to formyl-l-methionyl-l-leucyl-l-phenylalanine/cytochalasin B (fMLP/CB). Compounds **7**, **9**–**11**, **15**, and **17** inhibited fMLP/CB-induced elastase release with IC_50_ values ≤7.83 µg/mL. This investigation reveals that bioactive isolates (especially **6**, **7**, **14**, and **17**) could be further developed as potential candidates for the treatment or prevention of various inflammatory diseases.

## 1. Introduction

*Cnidium monnieri* (L.) Cusson (Umbelliferae) is an annual herb distributed in China, India, Russia, Korea, Mongolia, Vietnam, Europe, and North America [1,2]. Chromones [3,4], coumarins [5,6,7,8,9], benzofurans [10], and monoterpenoids [11], and their derivatives were isolated from this plant in previous studies. Many of these compounds were found to exhibit antidermatophytic [9], anti-scratching [5], and cytotoxic [8] activities. Granule proteases (e.g., elastase, cathepsin G, and proteinase-3) and reactive oxygen species (ROS) (e.g., superoxide anion (O_2_^•−^) and hydrogen peroxide) produced by human neutrophils are involved in the pathogenesis of a variety of inflammatory diseases.

**Figure 1:**
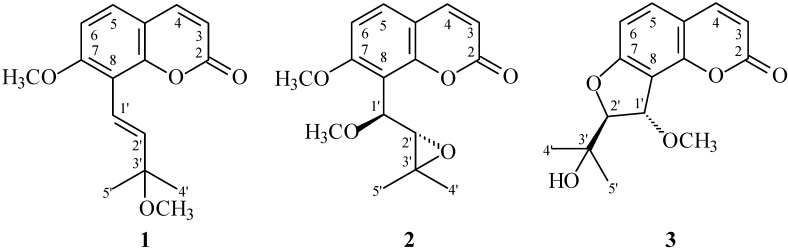
The chemical structures of new compounds **1**–**3** isolated from *C. monnieri*.

**Figure 2:**
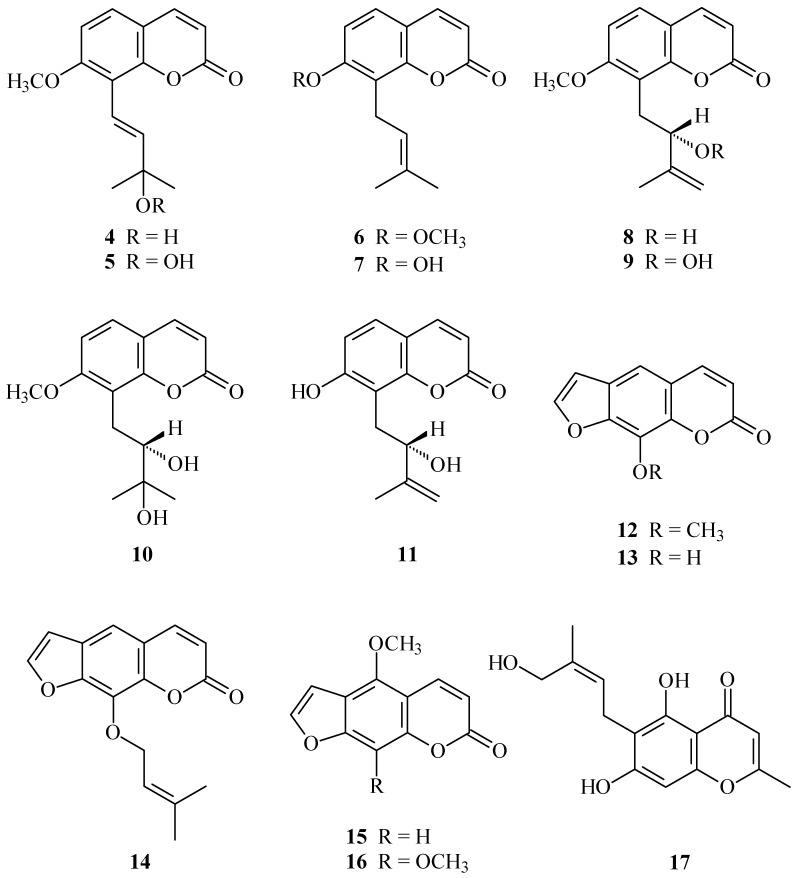
The chemical structures of known compounds **4**–**17** isolated from *C. monnieri*.

In our studies on the anti-inflammatory constituents of Formosan plants, many species have been screened for *in vitro* inhibitory activity on neutrophil pro-inflammatory responses, and *C. monnieri* has been found to be an active species. The MeOH extract of the fruits of *C. monnieri* showed potent inhibitory effects on superoxide anion generation and elastase release by human neutrophils in response to formyl-l-methionyl-l-leucyl-l-phenylalanine/cytochalasin B (fMLP/CB). Figure 1 illustrates the structures of three new coumarins, 3'-*O*-methylmurraol (**1**), *rel*-(1'*S*,2'*S*)-1'-*O*-methylphlojodicarpin (**2**), and (1'*S*,2'*S*)-1'-*O*-methylvaginol (**3**). Fourteen known compounds (**4**–**17**), have been isolated and identified from the fruits of *C. monnieri* and their structures are depicted in Figure 2.

This paper describes the structural elucidation of the compounds numbered **1** through **3**, and the inhibitory activities of all isolates on superoxide generation and elastase release by neutrophils.

## 2. Results

Chromatographic purification of the EtOAc-soluble fraction of a MeOH extract of fruits of *C. monnieri* on a silica gel column and preparative thin-layer chromatography (TLC) afforded three new (**1**–**3**) and 14 known compounds (**4**–**17**).

**Figure 3:**
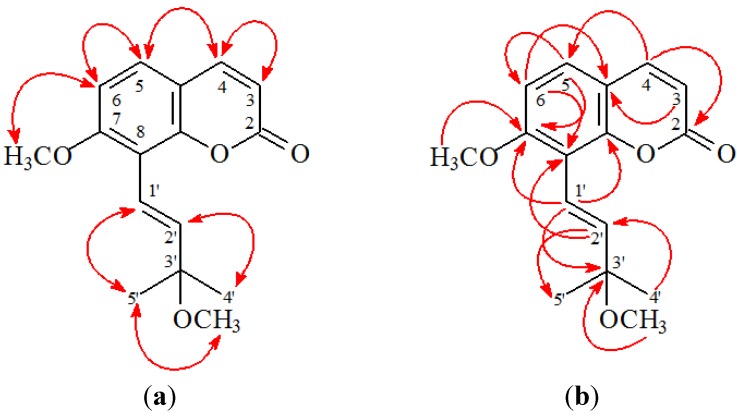
Key NOESY (**a**) and HMBC (**b**) correlations of **1**.

3'-*O*-Methylmurraol (**1**) was isolated as colorless prism with molecular formula C_16_H_18_O_4_ as determined by positive-ion HRESIMS, showing an [M + Na]^+^ ion at *m*/*z* 297.1105 (calcd for C_16_H_18_O_4_Na, 297.1103). The presence of a carbonyl group was revealed by a band at 1727 cm^−1^ in the IR spectrum, and was confirmed by the resonance at δ 160.9 in the ^13^C-NMR spectrum. The ^1^H-NMR spectrum of **1** showed the presence of an (*E*)-3-methoxy-3-methylbut-1-enyl group [δ 1.42 (6H, s, H-4', H-5'), 3.28 (3H, s, OMe-3'), 6.79 (1H, d, *J* = 16.0 Hz, H-2'), 6.83 (1H, d, *J* = 16.0 Hz, H-1')], a methoxy group [δ 3.95 (3H, s, OMe-7)], an AB spin system [δ 6.88 (1H, d, *J* = 9.0 Hz, H-6) and 7.31 (1H, d, *J* = 9.0 Hz, H-5)], and the typical H*-*3 and H*-*4 protons of the coumarin nucleus [δ 6.27, 7.63 (each 1H, each d, *J* = 9.5 Hz, H*-*3 and H-4)]. The ^1^H-NMR data of **1** was similar to those of murraol [12,13], except that the 3'-methoxy group [δ 3.28 (3H, s)] of **1** replaced the 3'-hydroxy group of murraol. This was supported by HMBC correlation observed between OMe-3' (δ 3.28) and C-3' (δ 75.9), and by NOESY correlations observed between OMe-3' (δ 3.28) and H-4'/H-5' (δ 1.42). The full assignment of ^1^H- and ^13^C-NMR resonances was confirmed by ^1^H-^1^H COSY, NOESY (Figure 3), DEPT, HSQC, and HMBC (Figure 3) techniques. According to the evidence above, the structure of **1** was elucidated as (*E*)-7-methoxy-8-(3-methoxy-3-methylbut-1-enyl)-2*H*-chromen-2-one, named 3'-*O*-methylmurraol.

**Figure 4:**
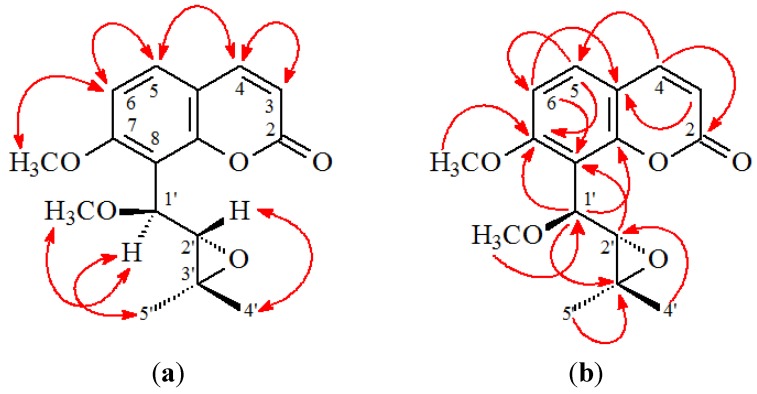
Key NOESY (**a**) and HMBC (**b**) correlations of **2**.

**Figure 5:**
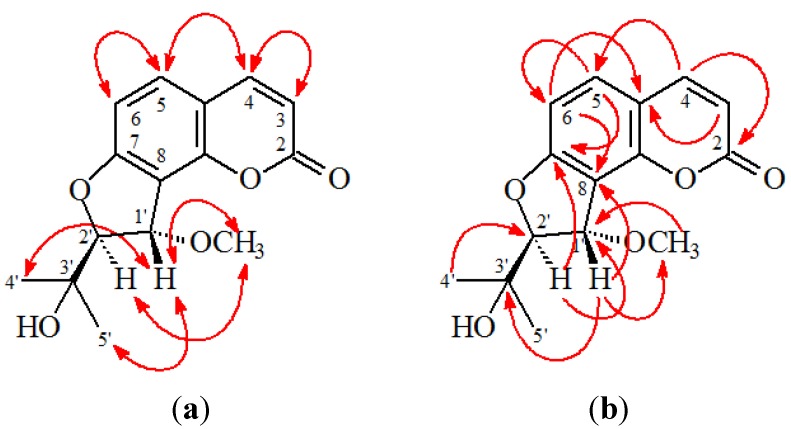
Key NOESY (**a**) and HMBC (**b**) correlations of **3**.

*rel*-(1'*S*,2'*S*)-1'-*O*-Methylphlojodicarpin (**2**) was obtained as optically active, colorless needles ([α]_D_^25^ = −28.4). Its molecular formula, C_16_H_18_O_5_, was determined on the basis of the positive HRESIMS at *m*/*z* 313.1050 [M + Na]^+^ (calcd 313.1052) and supported by the ^1^H-, ^13^C-, and DEPT NMR data. The presence of a carbonyl group was revealed by a band at 1733 cm^−1^ in the IR spectrum, and was confirmed by the resonance at δ 160.2 in the ^13^C-NMR spectrum. The ^1^H-NMR data of **2** were similar to phlojodicarpin [14], except that the 1'-methoxy group [δ 3.48 (3H, s)] of **2** replaced OH-1' of phlojodicarpin [14]. This was supported by the NOESY correlations between OMe-1' (δ 3.48) and H-1' (δ 4.84), and by the HMBC correlation between OMe-1' (δ 3.48) and C-1' (δ 75.1) of **2**. In addition, the *rel*-(1'*S*,2'*S*)-configuration of **2** was established by the following evidences: (a) The larger coupling constant (*J* = 7.0 Hz) between H_α_-1' and H_β_-2' of **2** was similar to that (*J* = 6.9 Hz) of hydroxyosthole epoxide with *rel*-(1'*S*,2'*S*)-configuration [15], and different from that (*J* = 3.0 Hz) of phlojodicarpin [14]; (b) The NOESY correlations were observed between H_α_-1'/Me_α_-3' and H-2'/Me_β_-3' (Figure 3); (c) Compound **2** showed a laevorotatory optical activity with [α]_D_^25^ = −28.4 as in the cases of hydroxyosthole epoxide ([α]_D_^25^ = −26.7) [15]. Thus, the structure of **2** was elucidated as *rel*-(1'*S*,2'*S*)-1'-*O*-methylphlojodicarpin. This structures was confirmed by the ^1^H-^1^H COSY, NOESY (Figure 4), DEPT, HSQC, and HMBC techniques (Figure 4).

(1'*S*,2'*S*)-1'-*O*-Methylvaginol (**3**) was isolated as an colorless prism ([α]_D_^25^ + 120.4). The molecular formula C_15_H_16_O_5_ was deduced from a sodium adduct ion at *m*/*z* 299.0892 [M + Na]^+^ (calcd 299.0895) in the HRESI mass spectrum. The presence of a carbonyl group was revealed by the band at 1727 cm^−1^ in the IR spectrum. The ^1^H- and ^13^C-NMR data of **3** were similar to those of vaginol [16], except that the 1'-methoxy group [δ_H_ 3.65 (3H, s, OMe-1'); δ_C_ 57.8 (OMe-1')] of **3** replaced 1'-hydroxy group of vaginol. This was supported by the HMBC correlation between OMe-1' (δ 3.65) and C-1' (δ 79.4) and by the NOESY correlations between OMe-1' (δ 3.65) and both H-1' (δ 5.23) and H-2' (δ 4.53). In addition, the 1'*S*,2'*S*-configuration of **3** was established by the following evidences: (a) The small coupling constant (*J* = 3.0 Hz) between H_β_-1' and H_α_-2' of **3** was similar to that (*J* = 3.6 Hz) of vaginol with 1'*S*,2'*S*-configuration [16], and different from that (*J* = 5.5 Hz) of vaginidiol with 1'*R*,2'*S*-configuration [16]; (b) The NOESY correlations were observed between OMe_α_-1'/H_α_-2', H_β_-1'/H-4', and H_β_-1'/H-5'; (c) Compound **3** showed a dextrorotatory optical activity with [α]_D_^25^ = +120.4, which was similar to vaginol ([α]_D_^2^^0^ = +119°) with 1'*S*,2'*S*-configuration [16], and different from vaginidiol ([α]_D_^2^^0^ = +223°) with 1'*R*,2'*S*-configuration [16]. According to the above data, the structure of **3** was elucidated as (1'*S*,2'*S*)-1'-*O*-methylvaginol. This was supported by ^1^H-^1^H COSY and NOESY (Figure 5) experiments, and ^13^C-NMR assignments were confirmed by DEPT, HSQC, and HMBC (Figure 5) techniques.

The known isolates were readily identified by a comparison of physical and spectroscopic data (UV, IR, ^1^H-NMR, [α]_D_, and MS) with corresponding authentic samples or literature values, and this included 13 coumarins, murraol (**4**) [12,13], peroxymurraol (**5**) [12,13], osthol (**6**) [12,17], osthenol (**7**) [18], auraptenol (**8**) [6,19], peroxyauraptenol (**9**) [12] [13], meranzin hydrate (**10**) [12,13], demethylauraptenol (**11**) [20], xanthotoxin (**12**) [17,21], xanthotoxol (**13**) [17], imperatorin (**14**) [17], bergapten (**15**) [17,21], and isopimpinellin (**16**) [17,21], and a chromone, cnidimol A (**17**) [22].

**Table 1:** Inhibitory effects of compounds **1**–**17** from the fruits of *C. monnieri* on superoxide radical anion generation and elastase release by human neutrophils in response to fMet-Leu-Phe/cytochalasin B ^a^.

Compounds	Superoxide Anion	Elastase
	IC_50_ [µg/mL] ^b^ or (Inh %) ^c^	
3'-*O*-Methylmurraol (**1**)	6.24 ± 0.50 ^g^	(35.10 ± 5.71) ^f^
1'-*O*-Methylphlojodicarpin (**2**)	(15.04 ± 1.26) ^g^	(6.02 ± 1.56) ^e^
1'-*O*-Methylvaginol (**3**)	(11.99 ± 2.14) ^f^	(6.42 ± 2.07) ^e^
Murraol (**4**)	2.83 ± 0.46 ^f^	(18.16 ± 5.62) ^e^
Peroxymurraol (**5**)	2.87 ± 0.11 ^g^	(5.78 ± 2.98)
Osthol (**6**)	0.005 ± 0.0002 ^f^	(31.96 ± 5.72) ^f^
Osthenol (**7**)	0.09 ± 0.01 ^g^	3.28 ± 0.90 ^g^
Auraptenol (**8**)	0.77 ± 0.11 ^e^	(22.46 ± 2.65) ^f^
Peroxyauraptenol (**9**)	0.41 ± 0.06 ^e^	7.83 ± 1.17 ^e^
Meranzin hydrate (**10**)	7.31 ±1.62 ^e^	4.21 ± 1.41 ^e^
Demethylauraptenol (**11**)	0.54 ± 0.05 ^g^	4.36 ± 1.67 ^f^
Xanthotoxin (**12**)	0.32 ± 0.13 ^e^	(14.81 ± 6.03)
Xanthotoxol (**13**)	(24.54 ± 2.47) ^g^	(42.94 ± 4.29) ^g^
Imperatorin (**14**)	0.07 ± 0.02 ^g^	(22.08 ± 2.93) ^f^
Bergapten (**15**)	0.36 ± 0.09 ^e^	4.62 ± 1.36 ^e^
Isopimpinellin (**16**)	2.75 ± 0.26	(8.97 ± 2.28) ^e^
Cnidimol A (**17**)	3.65 ± 0.41 ^g^	3.20 ± 0.16 ^g^
Diphenyleneiodonium ^d^	0.55 ± 0.20 ^g^	-
Phenylmethylsulfonyl fluoride ^d^	-	34.4 ± 5.5 ^g^

^a^ Results are presented as averages ± SEM (*n* = 4); ^b^ Concentration necessary for 50% inhibition (IC_50_). If IC_50_ value of compound was <10 µg/mL, it was displayed as IC_50_ [µg/mL]; ^c^ Percentage of inhibition (Inh %) at 10 µg/mL. If IC_50_ value of compound was ≥10 µg/mL, it was shown as (Inh %) at 10 µg/mL; ^d^ Diphenyleneiodonium and phenylmethylsulfonyl were used as positive controls for superoxide anion generation and elastase release, respectively; ^e^ *p* < 0.05 compared with the control; ^f^ *p* < 0.01 compared with the control; ^g^ *p* < 0.001 compared with the control.

The effects on neutrophil pro-inflammatory responses of compounds isolated from the fruits of *C. monnieri* were evaluated by suppressing fMet-Leu-Phe/cytochalasin B (fMLP/CB)-induced superoxide anion (O_2_^•−^) generation and elastase release by human neutrophils. The inhibitory activity data on neutrophil pro-inflammatory responses are summarized in Table 1. Diphenyleneiodonium and phenylmethylsulfonyl fluoride were used as positive controls for O_2_^•−^ generation and elastase release, respectively. From the results of our biological tests, the following conclusions can be drawn: (a) 3'-*O*-Methylmurraol (**1**), murraol (**4**), peroxymurraol (**5**), osthol (**6**), osthenol (**7**), auraptenol (**8**), peroxyauraptenol (**9**), meranzin hydrate (**10**), demethylauraptenol (**11**), xanthotoxin (**12**), imperatorin (**14**), bergapten (**15**), isopimpinellin (**16**), and cnidimol A (**17**) exhibited potent inhibition (IC_50_ ≤ 7.31 µg/mL) of superoxide anion (O_2_^•−^) generation by human neutrophils in response to fMLP/CB; (b) Osthenol (**7**), peroxyauraptenol (**9**), meranzin hydrate (**10**), demethylauraptenol (**11**), bergapten (**15**), and cnidimol A (**17**) exhibited potent inhibition (IC_50_ ≤ 7.83 µg/mL) against fMLP-induced elastase release; (c) Among the furocoumarin analogues (**12**–**16**), imperatorin (**14**) (with a 9-isoprenyloxy group) exhibited more effective inhibition than its analogues, xanthotoxin (**12**) (with a 9-methoxy group), xanthotoxol (**13**) (with a 9-hydroxy group), bergapten (**15**) (with a 5-methoxy group), and isopimpinellin (**16**) (with 5,9-dimethoxy groups) against fMLP-induced O_2_^•−^ generation; (d) Among the coumarins with 7,8-disubstituents (**1**–**11**), osthol (**6**) and osthenol (**7**) with 8-isoprenyl group exhibited more effective inhibition than its analogues (**1**–**5** and **8**–**11**) against fMLP-induced O_2_^•−^ generation; (e) Osthol (**6**) was the most effective among these compounds, with IC_50_ value of 0.005 ± 0.0002 µg/mL against fMLP-induced superoxide anion generation; (f) Cnidimol A (**17**) exhibited the most effective among the isolates, with IC_50_ value of 3.20 ± 0.16 µg/mL against fMLP-induced elastase release

## 3. Discussion

Seventeen compounds, including three new coumarins **1**–**3**, were isolated from the fruits of *C. monnieri*. Known compounds **5**, **7**, and **9** were obtained from this plant for the first time. The structures of these compounds were established on the basis of spectroscopic data. Further discovery of new coumarins from the genus *Cnidium* may not only provide more structure-activity data of coumarins, but may also contribute to enhancing our understanding of the taxonomy and evolution of the genus *Cnidium*.

Reactive oxygen species (ROS) (e.g., superoxide anion (O_2_^•−^), hydrogen peroxide) and granule proteases (e.g., elastase, cathepsin G) produced by human neutrophils contribute to the pathogenesis of inflammatory diseases. Inhibition of the inappropriate activation of neutrophils by drugs has been proposed as a way to ameliorate inflammatory diseases. Based on the results of our biological tests (Table 1), osthol (**6**), osthenol (**7**), and imperatorin (**14**) were the most effective among these compounds, with IC_50_ values of 0.005 ± 0.0002, 0.09 ± 0.01, and 0.07 ± 0.02 µg/mL, respectively, against fMLP-induced superoxide anion generation. Osthenol (**7**) and cnidimol A (**17**) exhibited the most effective among the isolates, with IC_50_ values of 3.28 ± 0.90 and 3.20 ± 0.16 µg/mL, respectively, against fMLP-induced elastase release. Compounds **6**, **7**, **14**, and **17** had been tested for their cytotoxicity on the NIH3T3 cell, where **6**, **7**, **14**, and **17** showed no significant activities with ED_50_ values >50 µg/mL. The above isolated compounds might support the traditional use of *C.* *monnieri* for the treatment of inflammatory processes. Thus, our study suggests *C.* *monnieri* and its isolates (especially **6**, **7**, **14**, and **17**) could be further developed as potential candidates for the treatment or prevention of various inflammatory diseases.

## 4. Experimental Section

### 4.1. Ethics Statement

Blood was taken from healthy human donors (20–30 years old) by venipuncture, using a protocol (No. 102-1595A3) approved by the Institutional Review Board at Chang Gung Memorial Hospital (Taoyuan, Taiwan). All donors gave written consent. The Medical Ethics Committee of Chang Gung Memorial Hospital approved this consent procedure.

### 4.2. General Experimental Procedures

Melting points were determined on a Yanaco micro-melting point apparatus (Kyoto, Japan) and were uncorrected. Optical rotations were measured using a Jasco DIP-370 polarimeter (Tokyo, Japan) in CHCl_3_. Ultraviolet (UV) spectra were obtained on a Jasco UV-240 spectrophotometer (Tokyo, Japan). Infrared (IR) spectra (neat or KBr) were recorded on a Perkin Elmer 2000 FT-IR spectrometer (Norwalk, CT, USA). Nuclear magnetic resonance (NMR) spectra, including correlation spectroscopy (COSY), nuclear Overhauser effect spectrometry (NOESY), heteronuclear multiple-bond correlation (HMBC), and heteronuclear single-quantum coherence (HSQC) experiments, were acquired using a Varian Inova 500 spectrometer operating at 500 MHz (^1^H) and 125 MHz (^13^C), respectively, with chemical shifts given in ppm (δ) using tetramethylsilane (TMS) as an internal standard. Electrospray ionisation (ESI) and high-resolution electrospray ionization (HRESI)-mass spectra were recorded on a Bruker APEX II or a VG Platform Electrospray ESI/MS mass spectrometer. Silica gel (70–230, 230–400 mesh, Merck) was used for column chromatography (CC). Silica gel 60 F-254 (Merck, Darmstadt, Germany) was used for thin-layer chromatography (TLC) and preparative thin-layer chromatography (PTLC).

### 4.3. Plant Material

The fruits of *C.* *monnieri* were collected from Yanpu, Pingtung County, Taiwan, in October 2010 and identified by Jih-Jung Chen. A voucher specimen (CM-201010) was deposited in the Department of Pharmacy, Tajen University, Pingtung, Taiwan.

### 4.4. Extraction and Isolation

The dried fruits (4.0 kg) of *C.* *monnieri* were extracted three times with MeOH (20 L each) for 3 days. The MeOH extracts were concentrated under reduced pressure at 35 °C, and the residue (420 g) was partitioned between EtOAc and H_2_O (1:1). The EtOAc layer was concentrated to give a residue (fraction A, 145 g). The water layer was further extracted with *n*-BuOH, and the *n*-BuOH-soluble part (fraction B, 132 g) and the water-solubles (fraction C, 128 g) were separated. Fraction A (110 g) was chromatographed on silica gel (70–230 mesh, 5.1 kg), eluting with *n*-hexane, gradually increasing the polarity with acetone to give 12 fractions: A1 (5 L, *n*-hexane), A2 (4 L, *n*-hexane/acetone, 99:1), A3 (4 L, *n*-hexane/acetone, 95:1), A4 (5 L, *n*-hexane/acetone, 90:1), A5 (4 L, *n*-hexane/acetone, 80:1), A6 (4 L, *n*-hexane/acetone, 70:1), A7 (4 L, *n*-hexane/acetone, 50:1), A8 (6 L, *n*-hexane/acetone, 30:1), A9 (4 L, *n*-hexane/acetone, 10:1), A10 (5 L, *n*-hexane/acetone, 3:1), A11 (4 L, *n*-hexane/acetone, 1:1), A12 (5 L, acetone). Fraction A3 (9.8 g) was washed with MeOH and filtered to yield **6** (325 mg) after recrystallization (*n*-hexane/EtOAc, 2:1). The filtrate was chromatographed on silica gel (230–400 mesh) eluting with *n*-hexane/EtOAc (10:1–0:1) to give 11 fractions (each 750 mL, A3-1–A3-11). Fraction A3-2 (95 mg) was purified by preparative TLC (silica gel, *n*-hexane/EtOAc, 9:5) to obtain **15** (4.3 mg) (*R_f_* = 0.71). Fraction A3-4 (98 mg) was purified by preparative TLC (silica gel, *n*-hexane/CHCl_3_, 13:7) to afford **12** (3.8 mg) (*R_f_* = 0.32). Fraction A4 (9.3 g) was chromatographed further on silica gel (230–400 mesh, 455 g) eluting with *n*-hexane/acetone (10:1–0:1) to give 12 fractions (each 800 mL, A4-1–A4-12). Fraction A4-2 (585 mg) was purified by CC (silica gel, hexane/acetone, 8:1–0:1) to afford 8 subfractions (each 250 mL, A4-2-1–A4-2-8). Fraction A4-2-3 (82 mg) was purified by preparative TLC (silica gel, *n*-hexane/EtOAc, 3:1) to obtain **6** (3.8 mg) (*R_f_* = 0.50) and **14** (4.1 mg) (*R_f_* = 0.38). Fraction A4-3 (136 mg) was purified by preparative TLC (silica gel, CHCl_3_/EtOAc, 30:1) to afford **15** (3.2 mg) (*R_f_* = 0.66) and **16** (3.5 mg) (*R_f_* = 0.49). Fraction A4-5 (125 mg) was purified by preparative TLC (silica gel, CH_2_Cl_2_/acetone, 50:1) to yield **9** (3.5 mg) (*R_f_* = 0.22). Fraction A5 (8.5 g) was chromatographed further on silica gel (230–400 mesh, 445 g) eluting with *n*-hexane/EtOAc (10:1–0:1) to give 10 fractions (each 850 mL, A5-1–A5-10). Fraction A5-2 (88 mg) was purified by preparative TLC (silica gel, *n*-CHCl_3_/EtOAc, 20:1) to obtain **7** (3.7 mg) (*R_f_* = 0.43). Fraction A5-3 (75 mg) was purified by preparative TLC (silica gel, *n*-hexane/EtOAc, 11:7) to obtain **2** (3.6 mg) (*R_f_* = 0.48). Fraction A5-4 (90 mg) was purified by preparative TLC (silica gel, *n*-hexane/EtOAc, 11:9) to give **8** (5.2 mg) (*R_f_* = 0.31). Fraction A5-5 (80 mg) was purified by preparative TLC (silica gel, *n*-hexane/EtOAc, 1:1) to afford **5** (3.7 mg) (*R_f_* = 0.36). Fraction A5-6 (105 mg) was purified by preparative TLC (silica gel, CH_2_Cl_2_/EtOAc, 2:1) to yield **13** (5.5 mg) (*R_f_* = 0.70). Fraction A6 (8.2 g) was chromatographed further on silica gel (230–400 mesh, 430 g) eluting with *n*-hexane/acetone (6:1–0:1) to give 8 fractions (each 850 mL, A6-1–A6-8). Fraction A6-2 (78 mg) was purified by preparative TLC (silica gel, CH_2_Cl_2_/EtOAc, 2:1) to obtain **3** (3.4 mg) (*R_f_* = 0.55). Fraction A6-3 (83 mg) was purified by preparative TLC (silica gel, CH_2_Cl_2_/EtOAc, 1:1) to obtain **1** (3.9 mg) (*R_f_* = 0.83). Fraction A8 (9.3 g) was chromatographed further on silica gel (230–400 mesh, 460 g) eluting with *n*-hexane/EtOAc (4:1–0:1) to give 9 fractions (each 900 mL, A8-1–A8-9). Fraction A8-2 (115 mg) was purified by preparative TLC (silica gel, CH_2_Cl_2_/EtOAc, 1:1) to afford **4** (4.3 mg) (*R_f_* = 0.62). Fraction A8-3 (86 mg) was purified by preparative TLC (silica gel, CH_2_Cl_2_/MeOH, 30:1) to yield **11** (3.9 mg) (*R_f_* = 0.43). Fraction A8-4 (86 mg) was purified by preparative TLC (silica gel, CH_2_Cl_2_/EtOAc, 1:1) to obtain **17** (3.2 mg) (*R_f_* = 0.61). Fraction A8-5 (102 mg) was purified by preparative TLC (silica gel, CH_2_Cl_2_/EtOAc, 1:1) to obtain **10** (3.8 mg) (*R_f_* = 0.19).

#### 4.4.1. 3'-*O*-Methylmurraol (**1**)

Colorless prisms (MeOH), m.p. 129–131 °C. UV (MeOH): λ_max_ (log ε) = 213 (4.24), 247 (3.76), 257 (3.77), 319 (4.12) nm. IR (KBr): υ_max_ = 1727 (C=O) cm^−1^. ^1^H-NMR (CDCl_3_, 500 MHz): δ = 1.42 (6H, s, H-4' and H-5'), 3.28 (3H, s, OMe-3'), 3.95 (3H, s, OMe-7), 6.27 (1H, d, *J* = 9.5 Hz, H-3), 6.79 (1H, d, *J* = 16.0 Hz, H-2'), 6.83 (1H, d, *J* = 16.0 Hz, H-1'), 6.88 (1H, d, *J* = 9.0 Hz, H-6), 7.31 (1H, d, *J* = 9.0 Hz, H-5), 7.63 (1H, d, *J* = 9.5 Hz, H-4). ^13^C-NMR (CDCl_3_, 125 MHz): δ = 25.9 (C-4'), 25.9 (C-5'), 50.7 (OMe-3'), 56.1 (OMe-7), 107.6 (C-6), 113.0 (C-4a), 113.2 (C-3), 113.8 (C-8), 117.1 (C-1'), 127.0 (C-5), 142.4 (C-2'), 143.8 (C-4), 152.6 (C-8a), 160.3 (C-7), 160.9 (C-2). ESI-MS: *m*/*z* = 297 [M + Na]^+^. HR-ESI-MS: *m*/*z* = 297.1105 [M + Na]^+^ (calcd for C_16_H_18_O_4_Na: 297.1103).

#### 4.4.2. *rel*-(1'*S*,2'*S*)-1'-*O*-Methylphlojodicarpin (**2**)

Colorless needles (CH_2_Cl_2_-MeOH); m.p. 141–143 °C. [α]_D_^25^: −28.4 (*c* 0.14, CHCl_3_). UV (MeOH): λ_max_ (log ε) = 219 (4.17), 244 (3.95), 253 (sh, 3.85), 318 (4.15) nm. IR (neat): υ_max_ 1733 (C=O) cm^−1^. ^1^H-NMR (CDCl_3_, 500 MHz): δ = 1.08 (3H, s, H-5'), 1.27 (3H, s, H-4'), 3.48 (3H, s, OMe-1'), 3.81 (1H, d, *J* = 7.0 Hz, H-2'), 3.94 (3H, s, OMe-7), 4.84 (1H, d, *J* = 7.0 Hz, H-1'), 6.27 (1H, d, *J* = 9.5 Hz, H-3), 6.91 (1H, d, *J* = 8.5 Hz, H-6), 7.44 (1H, d, *J* = 8.5 Hz, H-5), 7.64 (1H, d, *J* = 9.5 Hz, H-4). ^13^C-NMR (CDCl_3_, 125 MHz): δ = 19.4 (C-5'), 24.7 (C-4'), 56.8 (C-3'), 56.3 (OMe-7), 57.6 (OMe-1'), 65.6 (C-2'), 75.1 (C-1'), 108.0 (C-6), 113.1 (C-4a), 113.5 (C-3), 114.6 (C-8), 129.2 (C-5), 143.5 (C-4), 153.3 (C-8a), 160.2 (C-2), 160.7 (C-7). ESI-MS: *m*/*z* = 313 [M + Na]^+^. HR-ESI-MS: *m*/*z* = 313.1050 [M + Na]^+^ (calcd for C_16_H_18_O_5_Na: 313.1052).

#### 4.4.3. (1'*S*,2'*S*)-1'-*O*-Methylvaginol (**3**)

Colorless prisms (MeOH), m.p. 166–168 °C. UV (MeOH): λ_max_ (log ε) = 214 (4.31), 245 (sh, 3.86), 256 (sh, 3.80), 287 (sh, 3.91), 322 (4.18) nm. IR (KBr): υ_max_ = 3454 (OH), 1727 (C=O) cm^−1^. ^1^H-NMR (CDCl_3_, 400 MHz): δ = 1.26 (3H, s, H-4'), 1.33 (3H, s, H-5'), 1.72 (1H, br s, D_2_O exchangeable, OH-3'), 3.65 (3H, s, OMe-1'), 4.53 (1H, d, *J* = 3.0 Hz, H-2'), 5.23 (1H, d, *J* = 3.0 Hz, H-1'), 6.25 (1H, d, *J* = 9.5 Hz, H-3), 6.82 (1H, d, *J* = 8.5 Hz, H-6), 7.39 (1H, d, *J* = 8.5 Hz, H-5), 7.65 (1H, d, *J* = 9.5 Hz, H-4). ^13^C-NMR (CDCl_3_, 100 MHz): δ = 24.9 (C-4'), 25.3 (C-5'), 57.8 (OMe-1'), 79.4 (C-1'), 96.3 (C-2'), 107.5 (C-6), 112.6 (C-3), 113.2 (C-4a), 114.2 (C-8), 131.2 (C-5), 143.9 (C-4), 152.3 (C-8a), 160.5 (C-2), 164.6 (C-7). ESI-MS: *m*/*z* = 299 [M + Na]^+^. HR-ESI-MS: *m*/*z* = 299.0892 [M + Na]^+^ (calcd for C_15_H_16_O_5_Na: 299.0895).

#### 4.4.4. Supplementary Files

ESI-MS, HR-ESI-MS, ^1^H-NMR, and ^13^C-NMR spectra of three new compounds (**1**–**3**) are available as Supplementary Information.

### 4.5. Biological Assay

The effect of the isolated compounds on neutrophil pro-inflammatory response was evaluated by monitoring the inhibition of superoxide anion generation and elastase release in fMLP/CB-activated human neutrophils in a concentration-dependent manner. The purity of the tested compounds was >98% as identified by NMR and MS.

#### 4.5.1. Preparation of Human Neutrophils

Human neutrophils from venous blood of healthy, adult volunteers (20–28 years old) were isolated using a standard method of dextran sedimentation prior to centrifugation in a Ficoll Hypaque gradient and hypotonic lysis of erythrocytes [23]. Purified neutrophils containing >98% viable cells, as determined by the trypan blue exclusion method [24], were re-suspended in a calcium (Ca^2+^)-free HBSS buffer at pH 7.4 and were maintained at 4 °C prior to use.

#### 4.5.2. Measurement of Superoxide Anion Generation

The assay for measurement of superoxide anion generation was based on the SOD-inhibitable reduction of ferricytochrome *c* [25,26]. In brief, after supplementation with 0.5 mg/mL ferricytochrome *c* and 1 mM Ca^2+^, neutrophils (6 × 10^5^/mL) were equilibrated at 37 °C for 2 min and incubated with different concentrations (10–0.01 μg/mL) of compounds or DMSO (as control) for 5 min. Cells were incubated with cytochalasin B (1 μg/mL) for 3 min prior to the activation with 100 nM formyl-l-methionyl-l-leucyl-l-phenylalanine for 10 min. Changes in absorbance with the reduction of ferricytochrome *c* at 550 nm were continuously monitored in a double-beam, six-cell positioner spectrophotometer with constant stirring (Hitachi U-3010, Tokyo, Japan). Calculations were based on differences in the reactions with and without SOD (100 U/mL) divided by the extinction coefficient for the reduction of ferricytochrome *c* (ε = 21.1/mM/10 mm).

#### 4.5.3. Measurement of Elastase Release

Degranulation of azurophilic granules was determined by measuring elastase release as described previously [25,26]. Experiments were performed using MeO-Suc-Ala-Ala-Pro-Val-*p*-nitroanilide as the elastase substrate. Briefly, after supplementation with MeO-Suc-Ala-Ala-Pro-Val-*p*-nitroanilide (100 μM), neutrophils (6 × 10^5^/mL) were equilibrated at 37 °C for 2 min and incubated with compounds for 5 min. Cells were stimulated with fMLP (100 nM)/CB (0.5 µg/mL), and changes in absorbance at 405 nm were monitored continuously in order to assay elastase release. The results were expressed as the percent of elastase release in the fMLP/CB-activated, drug-free control system.

#### 4.5.4. Statistical Analysis

Results are expressed as the mean ± SEM, and comparisons were made using Student’s *t*-test. A probability of 0.05 or less was considered significant. The software SigmaPlot was used for the statistical analysis.

## 5. Conclusions

Seventeen compounds, including three new coumarins (**1**–**3**), were isolated from the fruits of *C. monnieri*. The structures of these compounds were established on the basis of spectroscopic data. Reactive oxygen species (ROS) (e.g., superoxide anion (O_2_^•−^), hydrogen peroxide) and granule proteases (e.g., elastase, cathepsin G) produced by human neutrophils contribute to the pathogenesis of inflammatory diseases. The effects on neutrophil pro-inflammatory responses of isolates were evaluated by suppressing fMLP/CB-induced O_2_^•−^ generation and elastase release by human neutrophils. The results of anti-inflammatory experiments indicate that compounds **1**, **4**–**12**, and **14**–**17** can significantly inhibit fMLP-induced O_2_^•−^ generation and/or elastase release. Osthol (**6**) and cnidimol A (**17**) were the most effective among the isolated compounds, with IC_50_ values of 0.005 ± 0.0002 and 3.20 ± 0.16 µg/mL, respectively, against fMLP-induced O_2_^•−^ generation and elastase release. Our study suggests *C. monnieri* and its isolates (especially **6**, **7**, **14**, and **17**) could be further developed as potential candidates for the treatment or prevention of various inflammatory diseases.

## Supplementary Files

Supplementary File 1 Supplementary Information (PDF, 1858 KB)
