# Supplementary material for: New Coumarins and Anti-Inflammatory Constituents from the Fruits of Cnidium monnieri
Source: Int J Mol Sci. 2014 May 28;15(6):9566–78. doi: 10.3390/ijms15069566 (PMC4100110; doi:10.3390/ijms15069566)

Supplementary Information

Figure S1. ESI-MS spectrum of **1**.

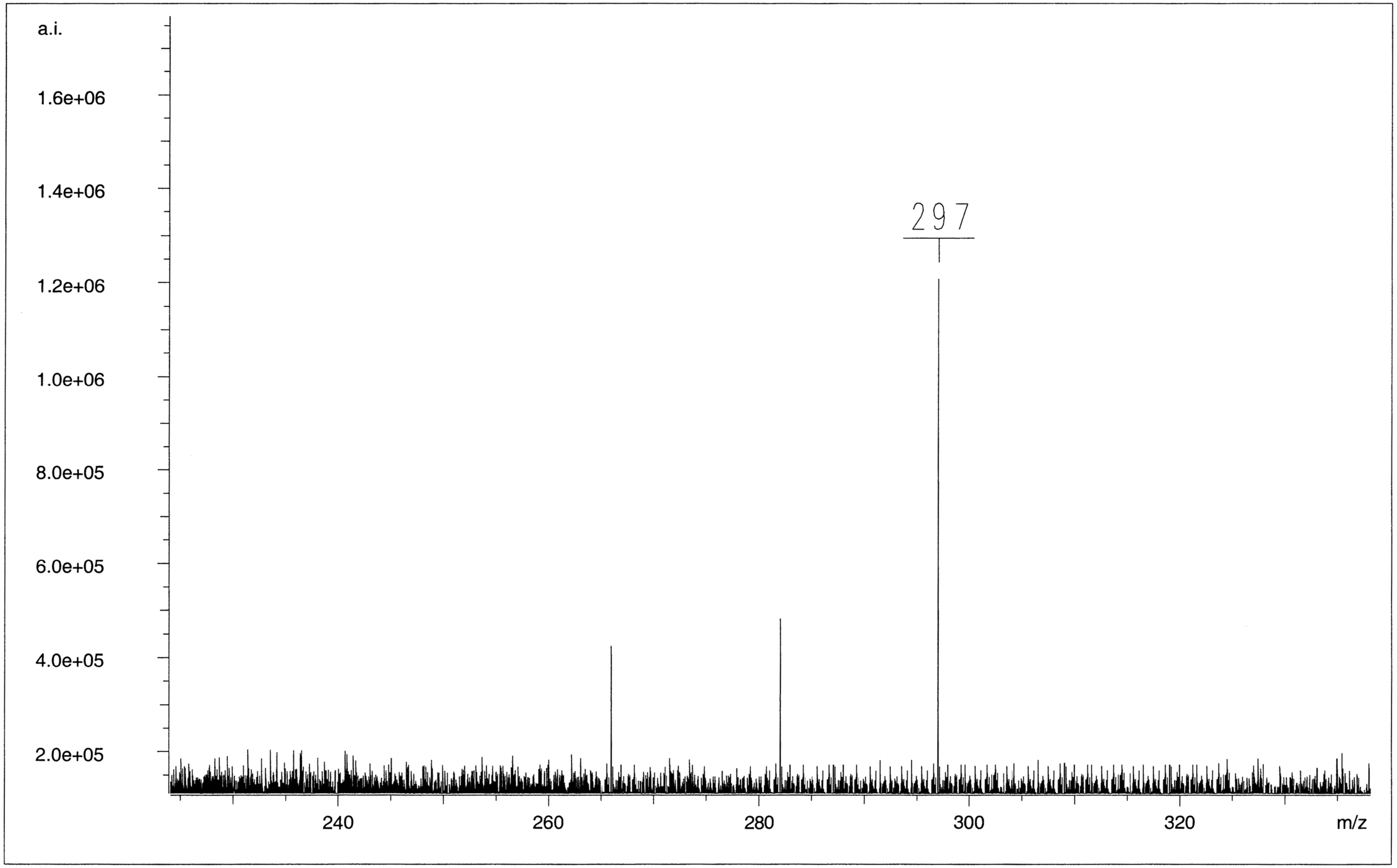

Figure S2. HR-ESI-MS spectrum of **1**.

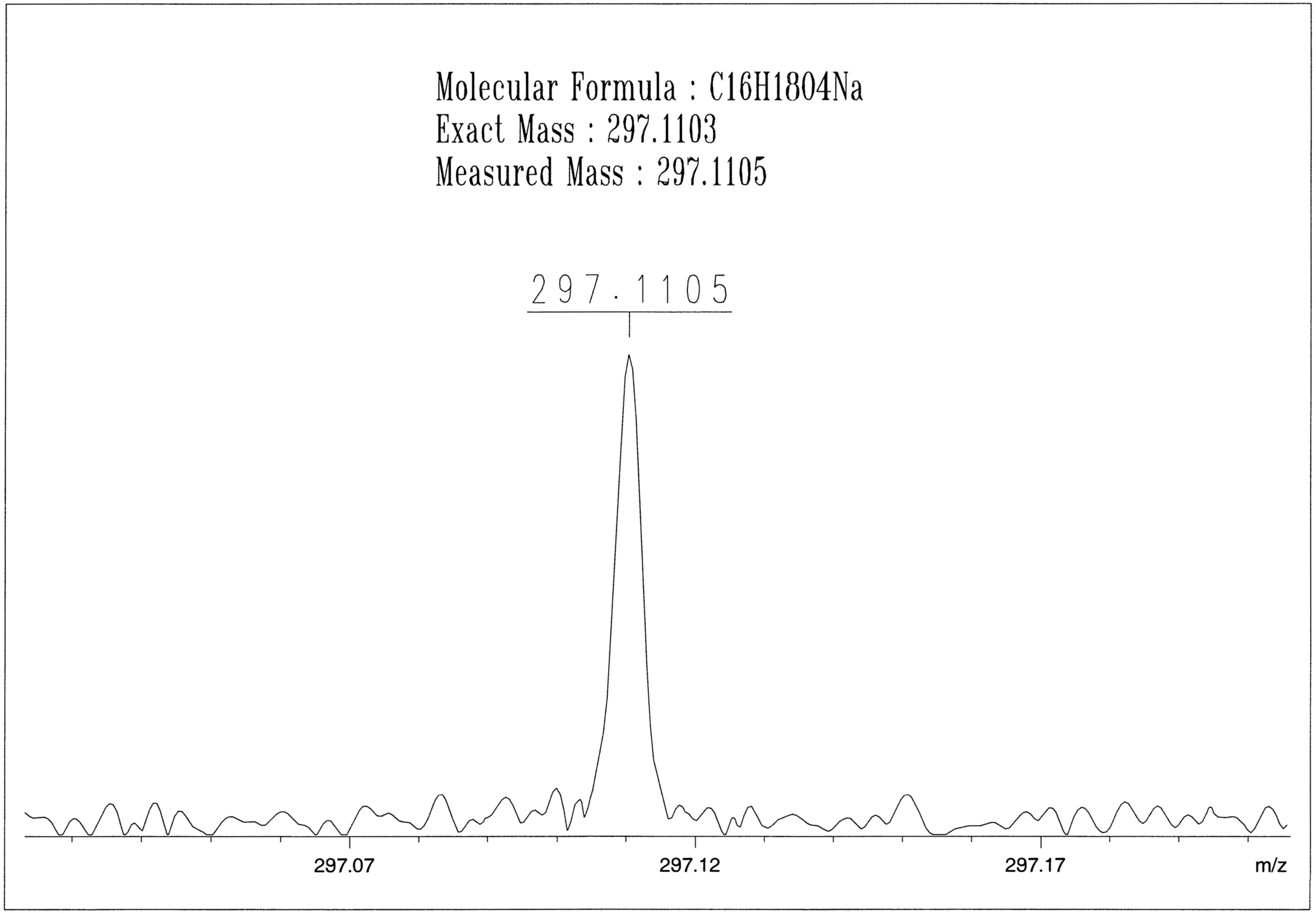

Figure S3. ^{1}H-NMR spectrum ($CDCl_3$, 500 MHz) of **1**.

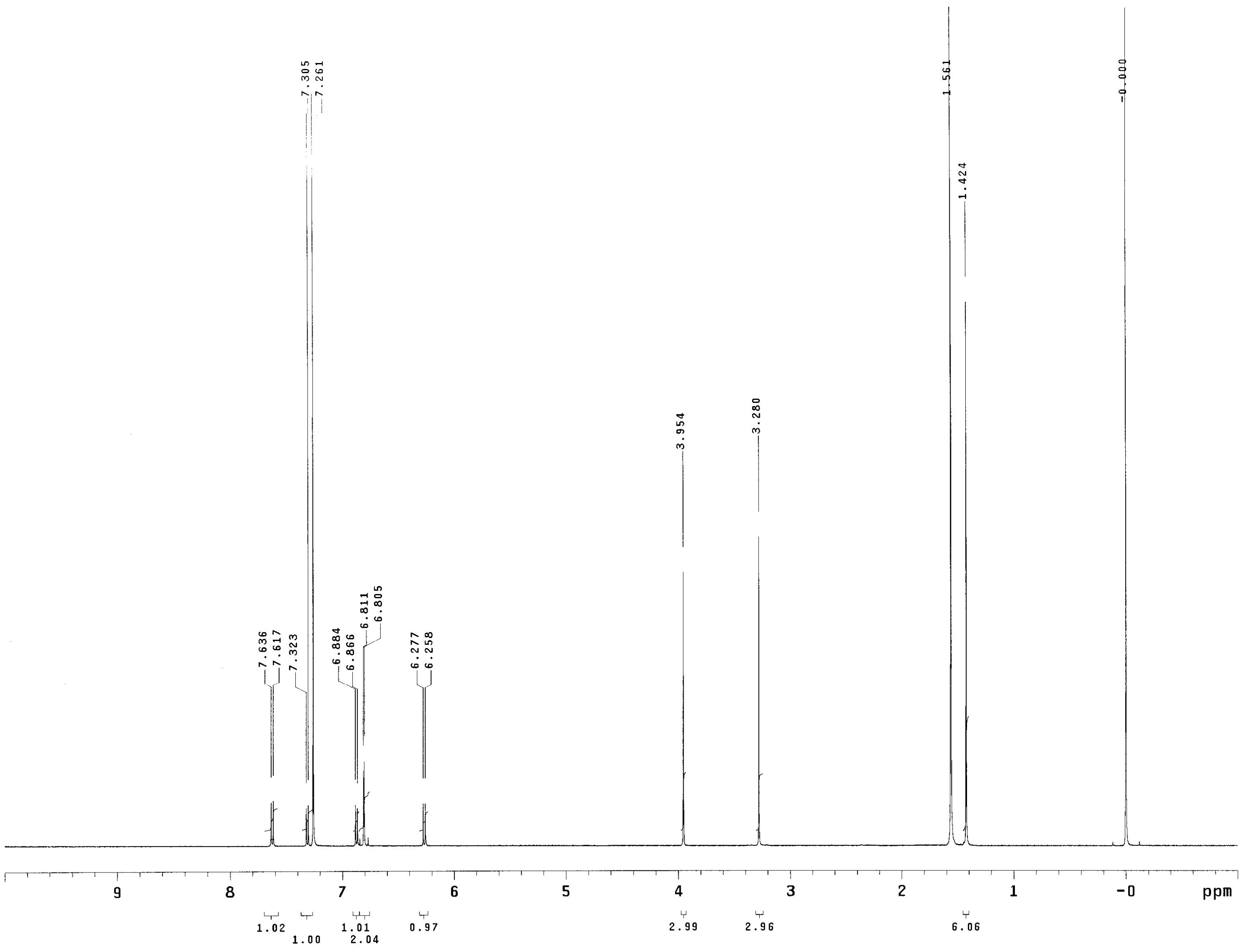

Figure S4. ^{13}C-NMR spectrum ($CDCl_3$, 125 MHz) of **1**.

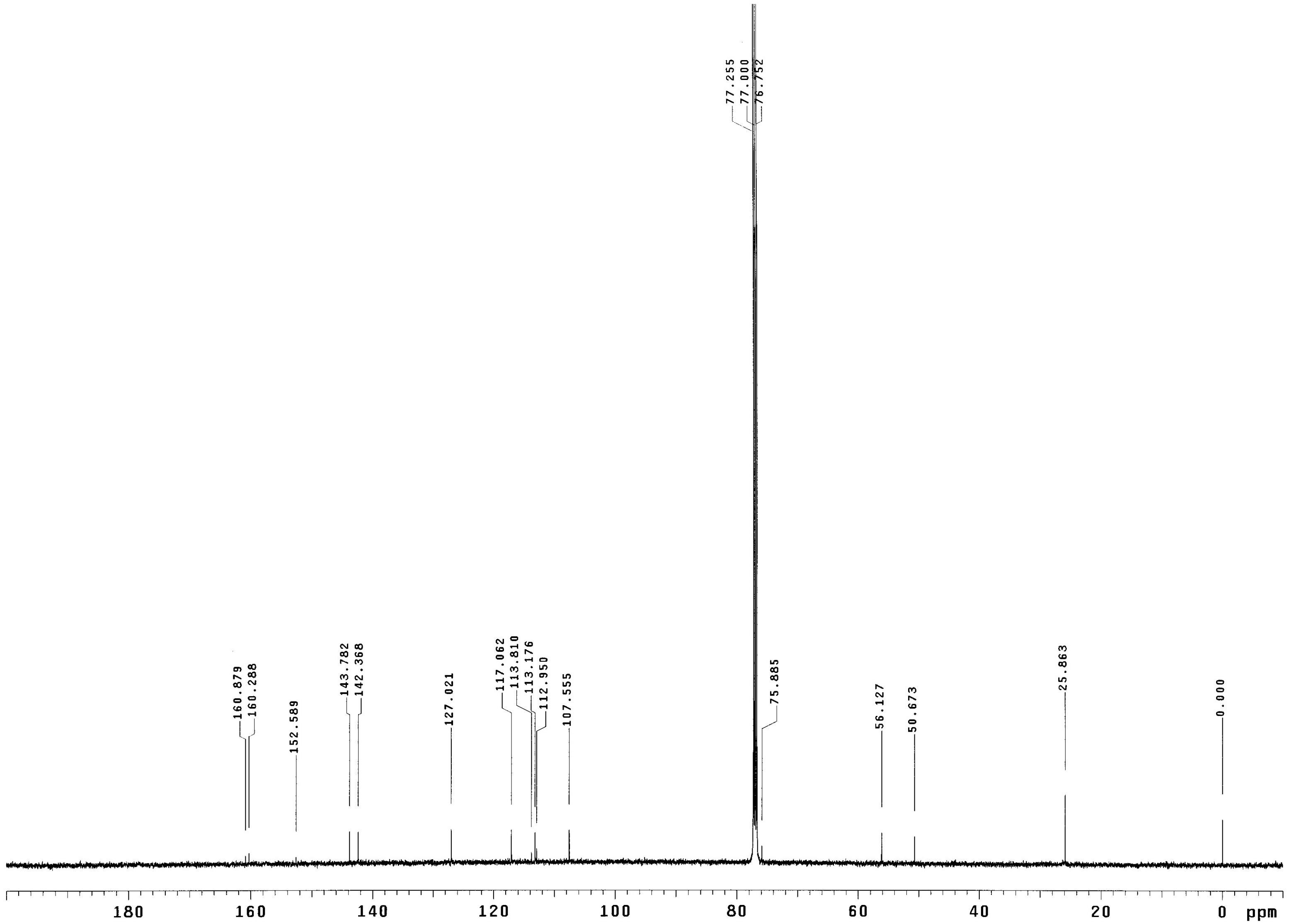

Figure S5. ESI-MS spectrum of **2**.

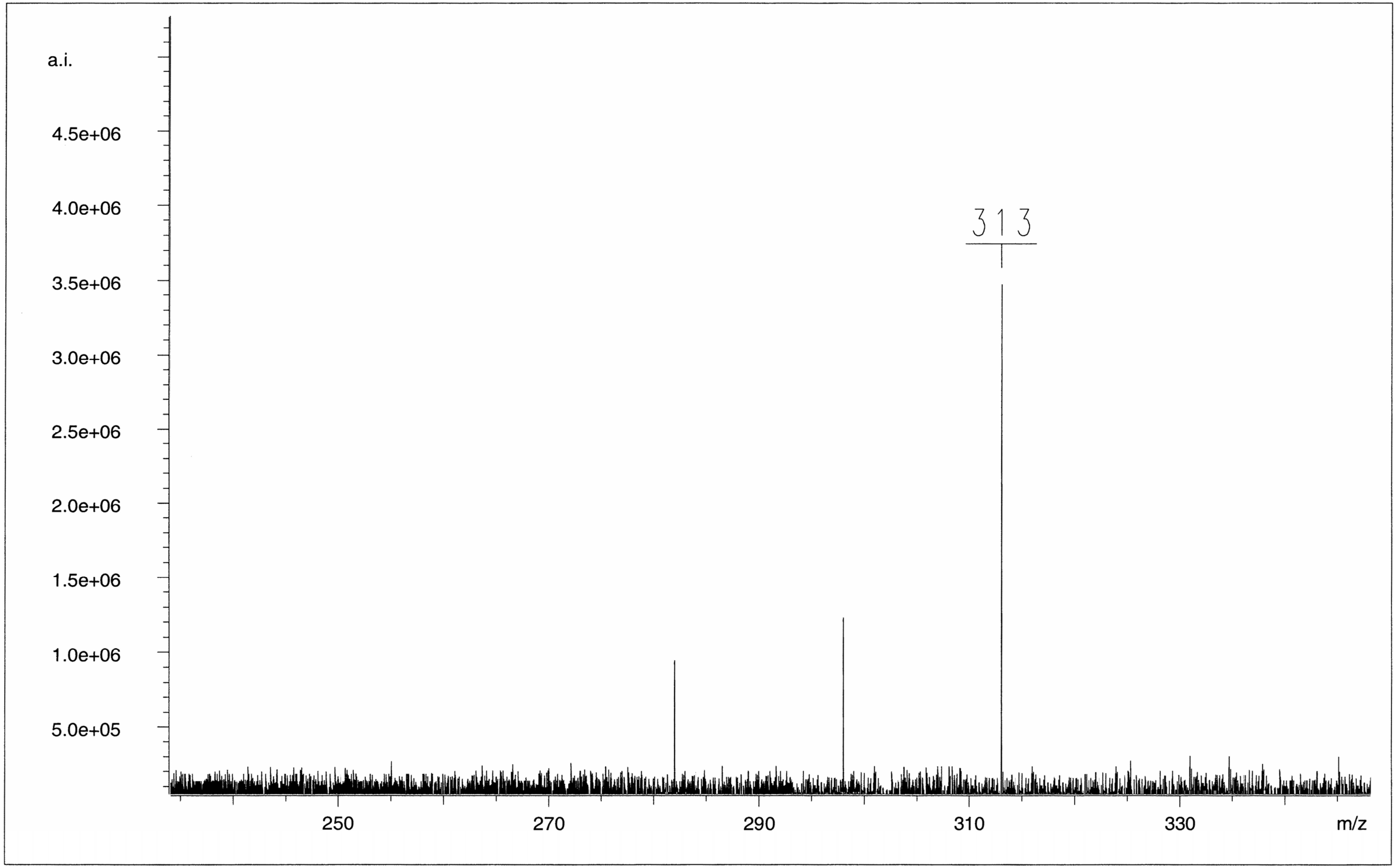

Figure S6. HR-ESI-MS spectrum of **2**.

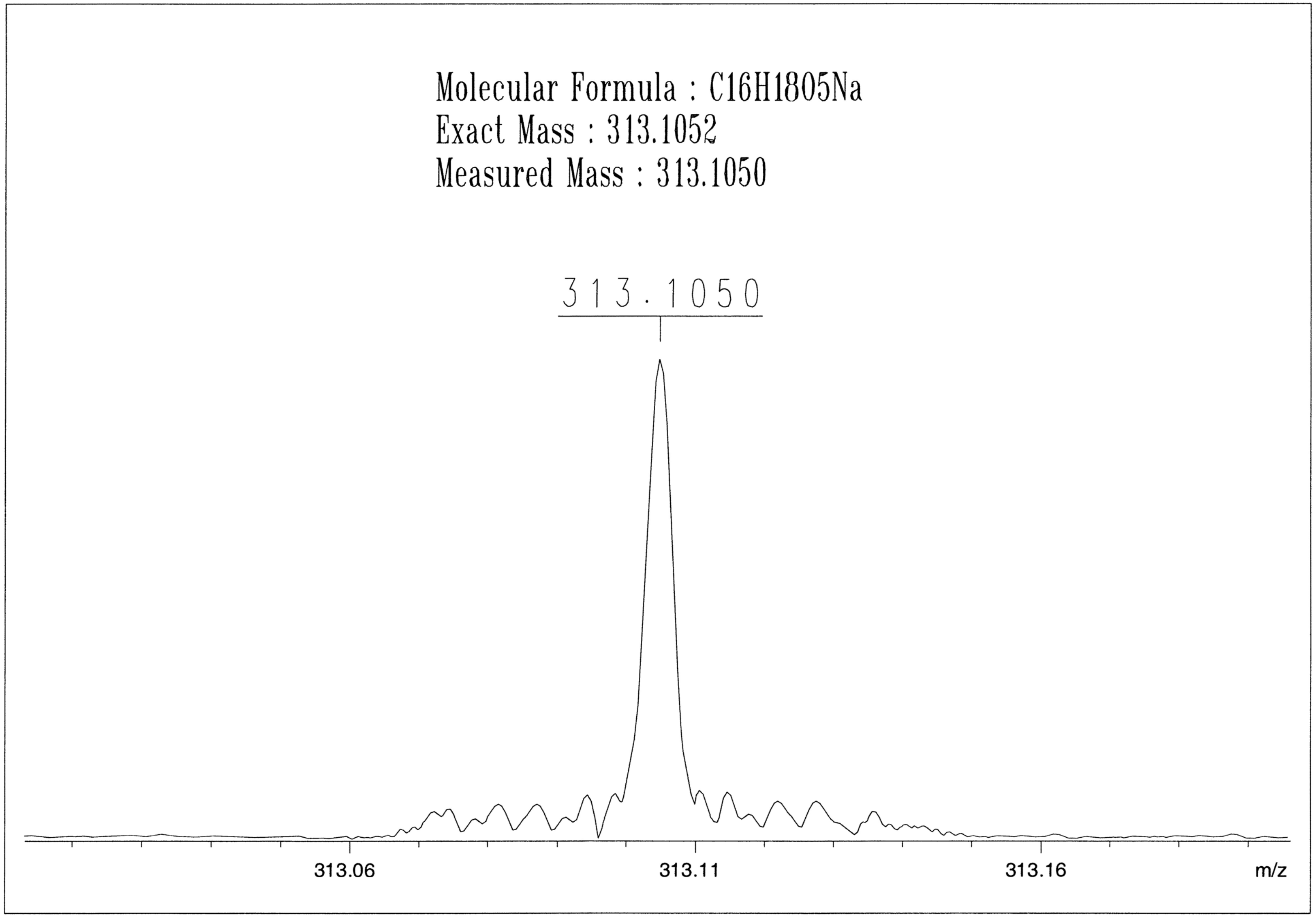

Figure S7. ^{1}H-NMR spectrum ($CDCl_3$, 500 MHz) of **2**.

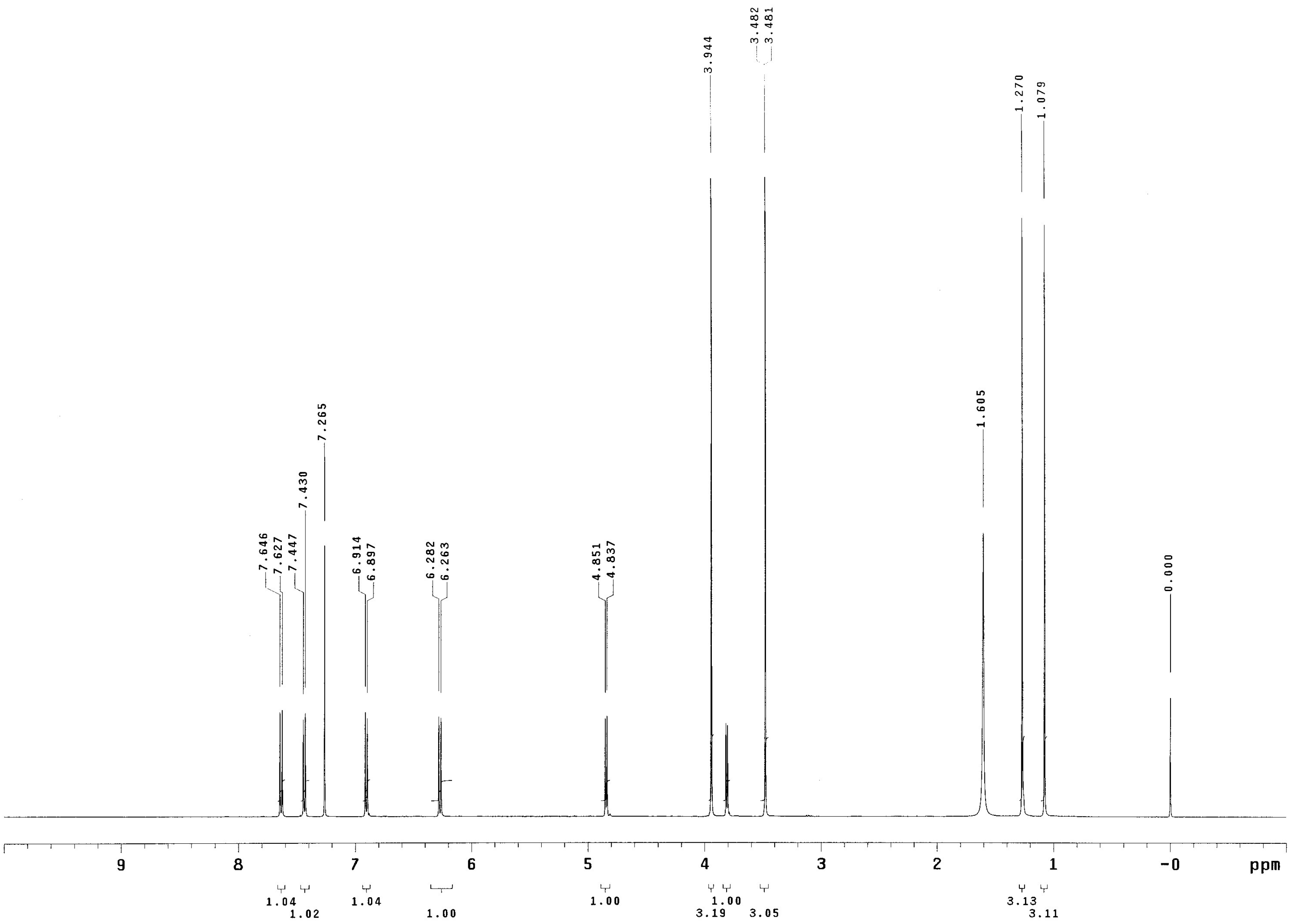

Figure S8. ^{13}C-NMR spectrum ($CDCl_3$, 125 MHz) of **2**.

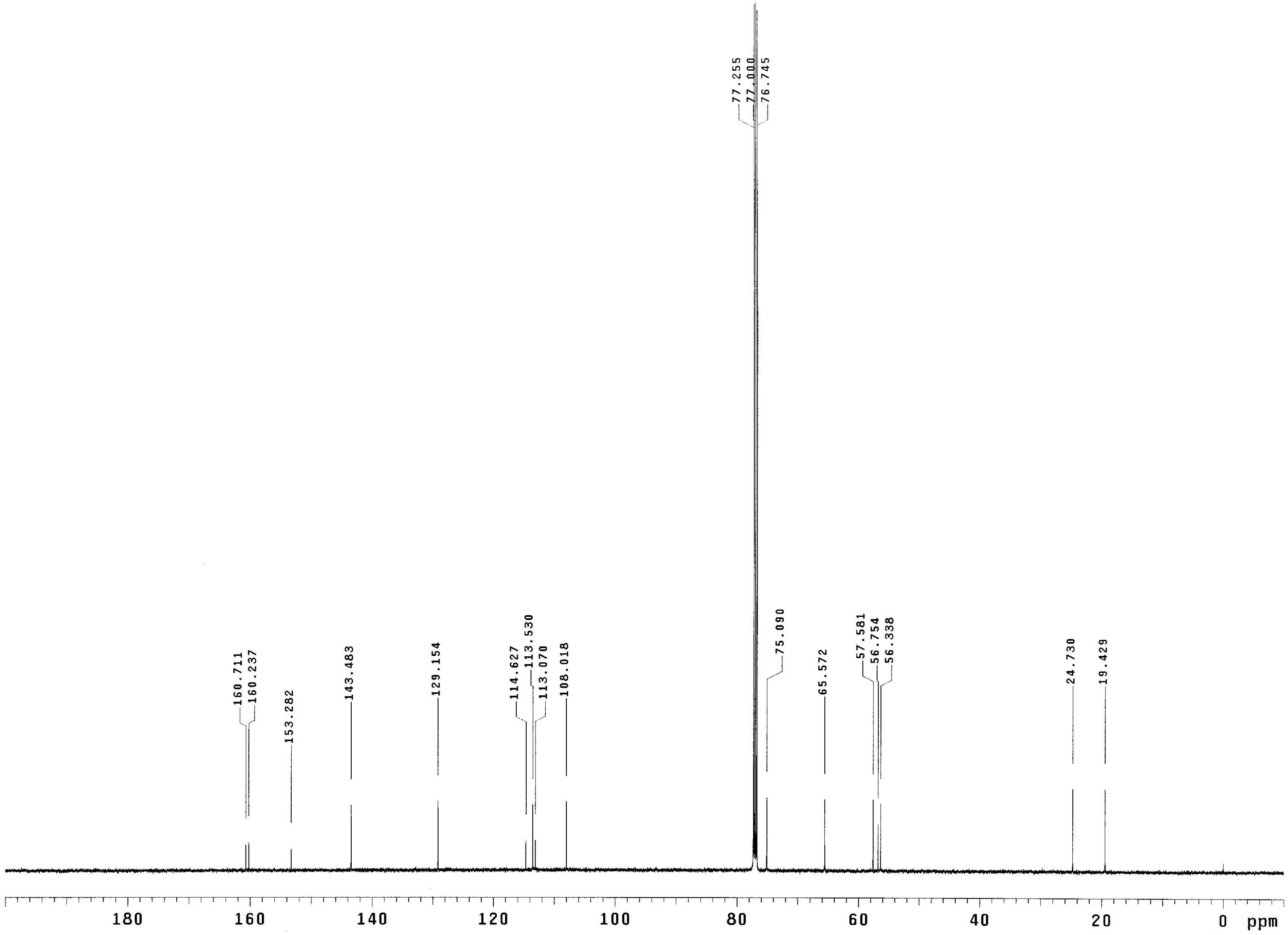

Figure S9. ESI-MS spectrum of **3**.

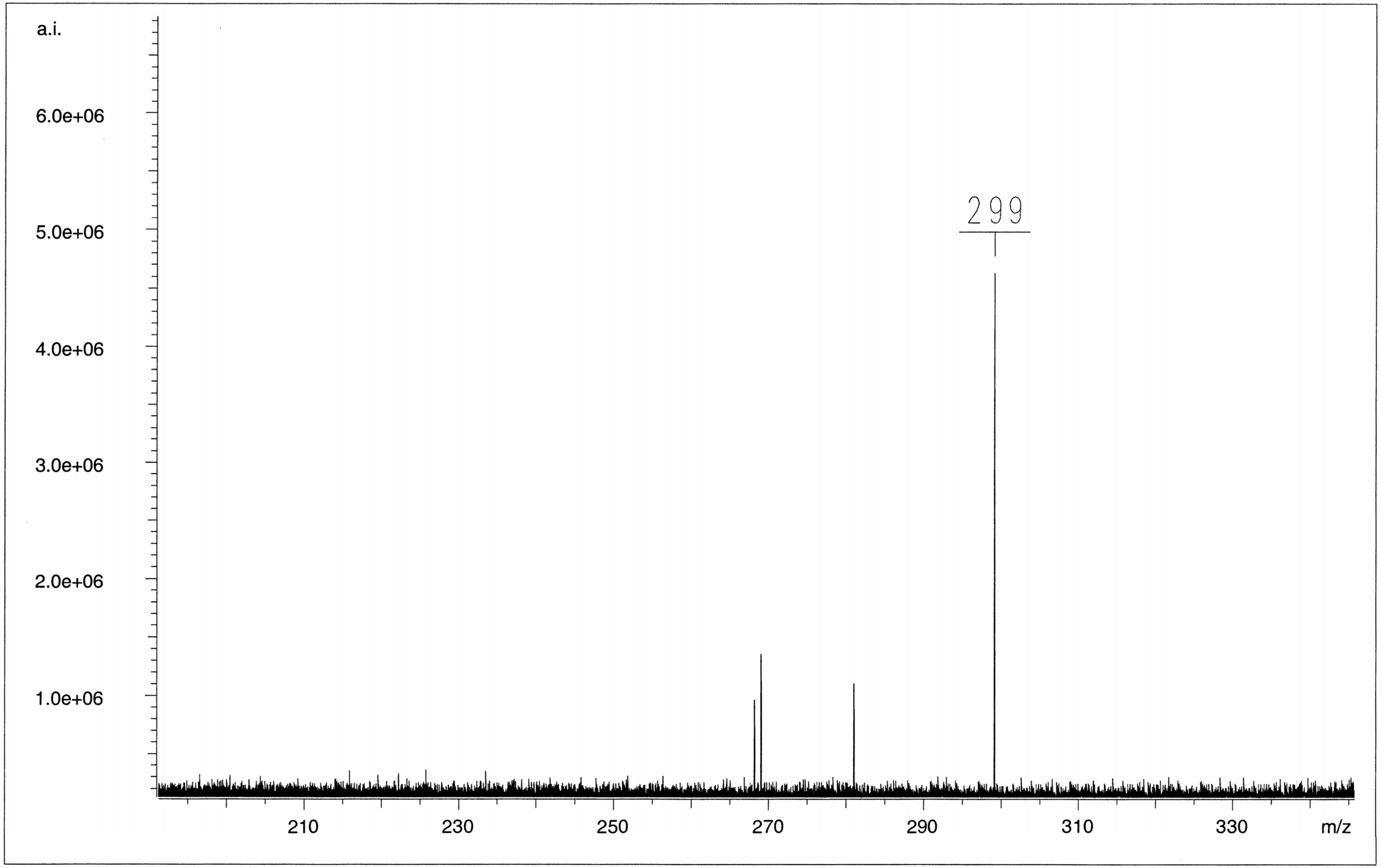

Figure S10. HR-ESI-MS spectrum of **3**.

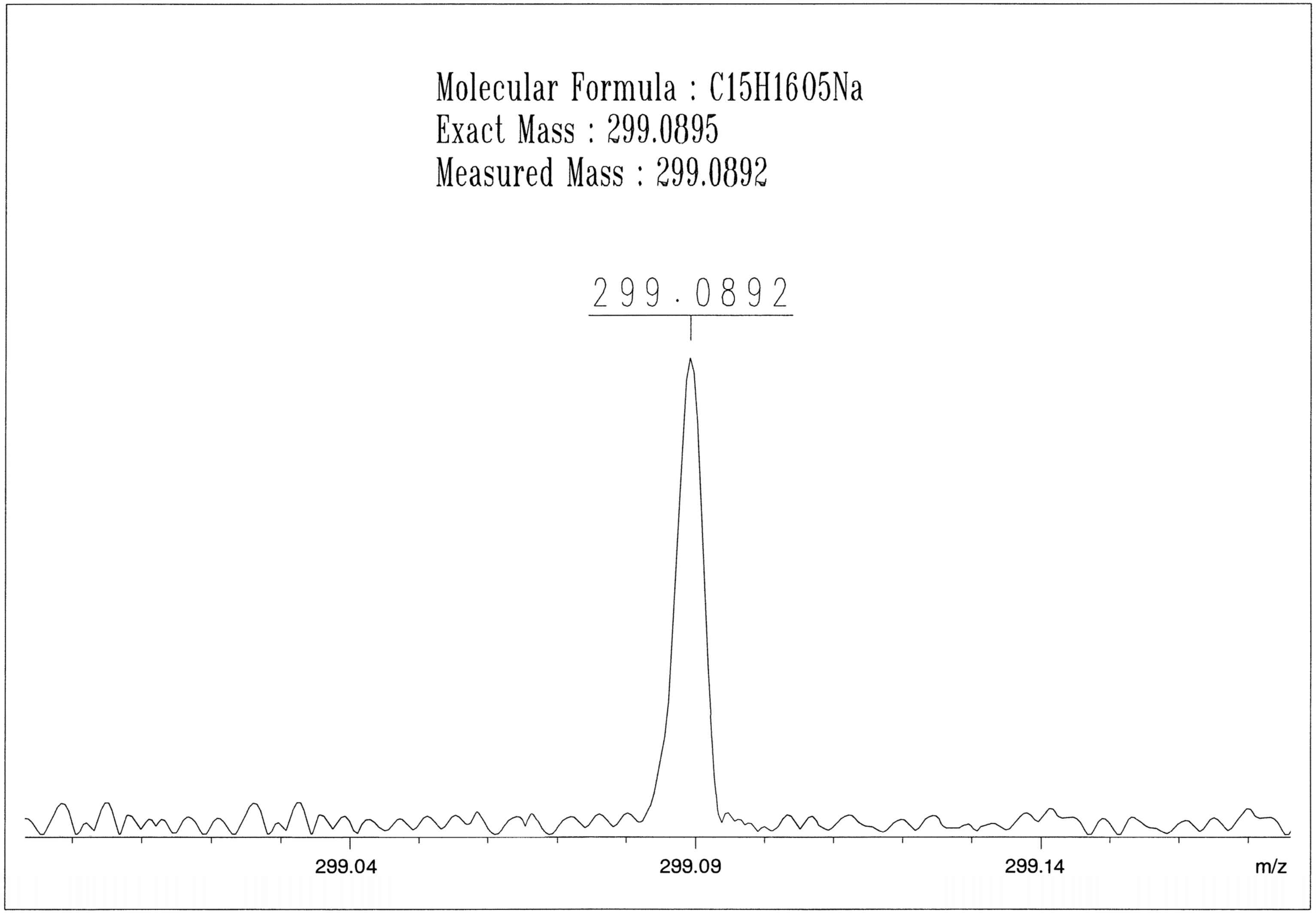

Figure S11. ^{1}H-NMR spectrum ($CDCl_3$, 500 MHz) of **3**.

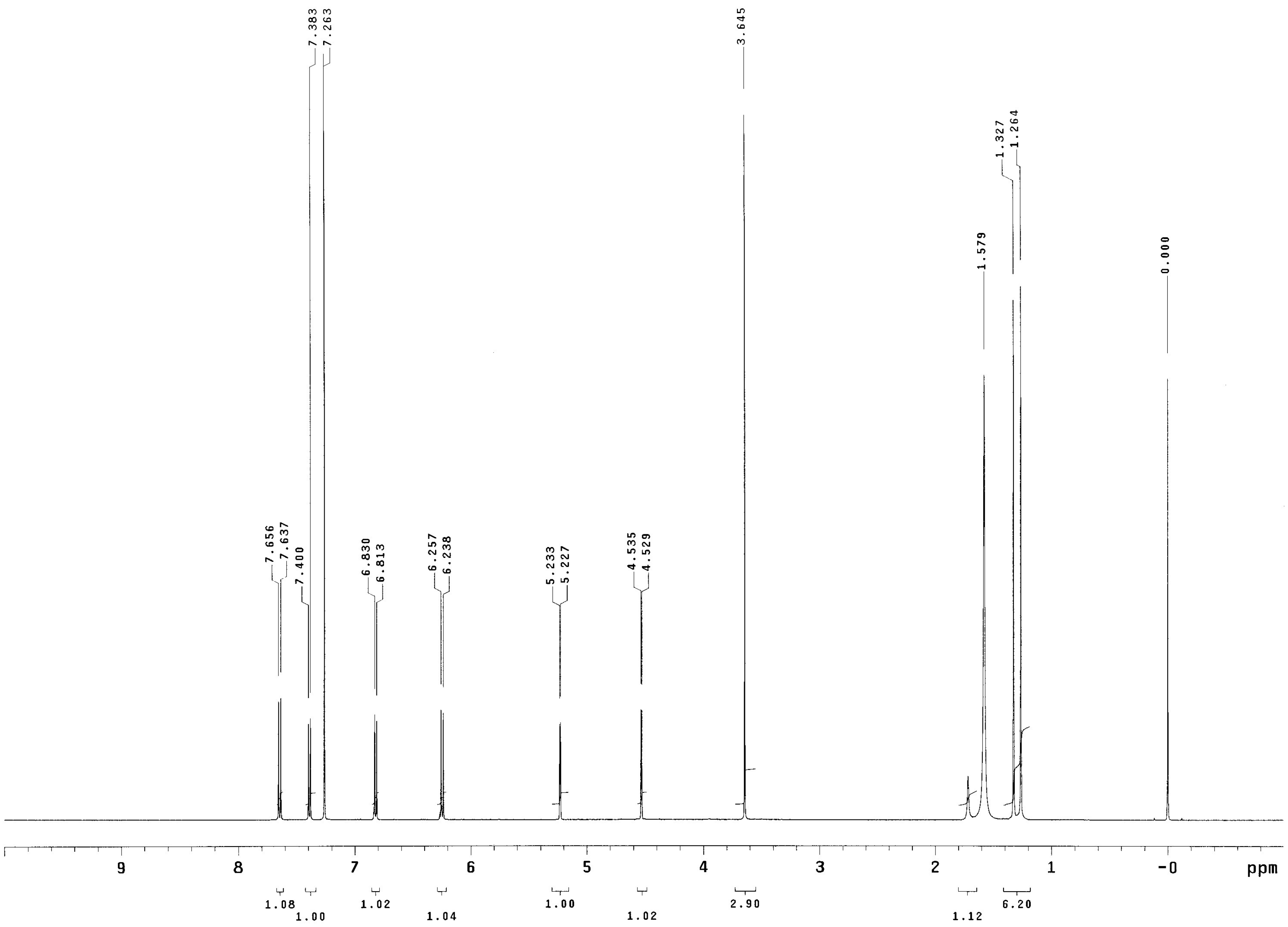

Figure S12. ^{13}C-NMR spectrum of **3** ($CDCl_3$, 125 MHz).

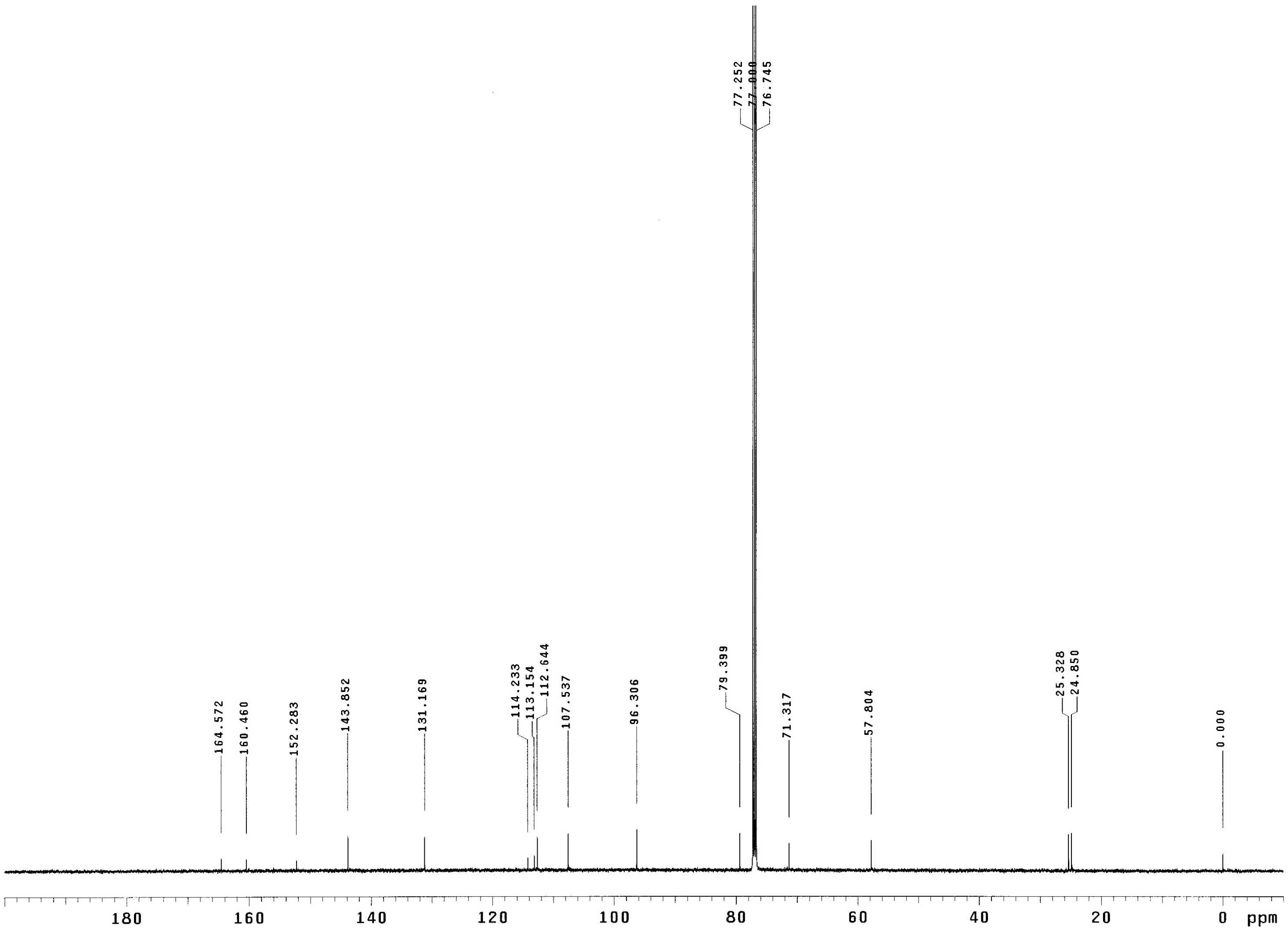

Supplement: Supplementary File 1 — Supplementary Information (PDF, 1858 KB) [file ijms-15-09566-s001.pdf]
